# Protective effect of *Cupressus sempervirens* extract against indomethacin-induced gastric ulcer in rats

**DOI:** 10.1515/intox-2015-0006

**Published:** 2015-03

**Authors:** Khaled M. M. Koriem, Islam B. Gad, Zayenab K. Nasiry

**Affiliations:** 1 Medical Physiology Department, Medical Research Division, National Research Centre, Dokki, Giza, Egypt; 2 Integrative Medicine Cluster, Advanced Medical and Dental Institute (AMDI), University Sains Malaysia (USM), Malaysia; 3 Department of Pathology, Faculty of Medicine, Ain Shams University, Egypt; 4 Faculty of Pharmacy, Cairo University, Egypt

**Keywords:** gastric ulcer, *Cupressus sempervirens*, Cupressuflavone, antioxidants, apoptosis

## Abstract

*Cupressus sempervirens (C. sempervirens)* belongs to the family Cupressaceae. It is widspread in Northern Africa, Greece, Turkey, North America, Cyprus and Syria. Cupressuflavone is the major ingredient of the plant leave extract. The aim of the present study was to evaluate the antiulcerogenic activity of the extract of *C. sempervirens* leaves in gastric ulcer tissues induced by indomethacin. The results of the present study revealed that indomethacin significantly decreased glutathione S-transferase (GST), glutathione peroxidase (GPx), catalase (CAT), reduced glutathione (GSH), glutathione reductase (GR) and superoxide dismutase (SOD) levels, while it increased significantly lipid peroxidation (MDA), nitric oxide (NO) and protein carbonyl (PC) levels in gastric tissue. Furthermore, indomethacin decreased *p53* expression, while it increased bcl-2 expression in gastric tissue. Pretreatment with 5%, 10% & 20% of the LD_50_ of *C. sempervirens* and cupressuflavone of indomethacin-treated rats restored all the above parameters to approach normal values. *C. sempervirens* at the highest dose was more effective than the two lower doses. *C. sempervirens* proved more potent than cupressuflavone. In conclusion, *C. sempervirens* exerted antiulcerogenic activity and the effect was dose-dependent and related to the cupressuflavone ingredient of the plant leave extract.

## Introduction

Gastric ulcer is a disease that affects a considerable number of people in the world and it is induced by several factors including stress, smoking, nutritional deficiencies and ingestion of non-steroidal anti-inflammatory drugs. Protection of the gastric mucosa involves factors such as acid-pepsin secretion, parietal cell activity, mucosal barrier, mucus secretion, blood flow, cell regeneration, and release of endogenous protective agents, especially prostaglandins and epidermal growth factors. Nonsteroidal anti-inflammatory drugs (NSAIDs) are used widely in the treatment of pain, fever and inflammation. However, these drugs have some side effects, especially in the gastrointestinal tract. Reactive oxygen species (ROS) play a role in gastric ulcer (Das *et al*., 1997). The role of ROS in the pathogenesis of acute experimental gastric lesions induced by stress NSAIDs is well-known (Das *et al*., 1997; Isenberg *et al*., 1995). Gastric damage may also be attributed to the presence of super-oxide anion (O_2_^–^). Much attention has been focused on oxygen-derived free radicals. Recent developments in biomedical science may involve some degenerative effects (Ames *et al*., 1993). The development of safe and effective drugs capable of preventing stomach damage induced by NSAIDs or other gastric damaging substances represents an important goal of medicinal research considering the large use of these drugs and the increased healthcare costs when peptic ulcer disease becomes a chronic condition. It is well established that natural products are an excellent source of chemical structures with a wide variety of biological activity, including gastro-protective properties.

Plant extracts are some of the most attractive sources for new drugs and have been shown to produce promising results in the treatment of gastric ulcer (Toma *et al*., 2002). *Cupressus sempervirens (C. sempervirens)* belongs to the family Cupressaceae. *C. sempervirens* is native to the north of Africa, Greece, Turkey, North America, Cyprus and Syria (Hortus, 1976). It is an ornamental tree up to 35 m tall, with a conic crown with level branch. The foliage grows in dense sprays. The leaves are scale-like shoots. The seed cones are ovoid or oblong with 10–14 scales, the male cones are 3–5 mm long (Vidakovic, 1991; Waisel & Epstein, 2000). *C. sempervirens* contains cupressuflavone as a major ingredient in addition to amenoflavone, rutin, quercetin, myricitrin flavonoids (Waisel & Epstein, 2000; Agrawal, 1989). It includes some phenolic compounds (anthocyanidin, catechine flavones, flavonols and isoflavones), tannins (ellagic acid, gallic acid, phenyl isopropanoids, caffeic acid, coumaric acid, ferulic acid), 6 lignans and catchol (Harborne, 1993).

*C. sempervirens* is used as antihelmintic, antipyretic, antirheumatic, antiseptic, astringent, balsamic, vasoconstrictive and anti-inflammatory agent (Kassem *et al*., 1991; Uncini *et al*., 2001; Said *et al*., 2002). It is used in the treatment of whooping cough, spitting up of blood, spasmodic cough, cold, flu and sore throats. *C. sempervirens* is applied externally as a lotion. It astringes varicose veins and hemorrhoids, tightening up blood vessels (Duke, 2002). A resin obtained from the tree has a positive action on slow-healing wounds (Chiej, 1984). There are many other uses of *C. sempervirens* for cosmetic and soap making purposes (Uphof, 1959; Usher, 1974).

The aim of the present study was to evaluate the antiulcerogenic activity of *C. sempervirens* leaves in gastric ulcer induced by indomethacin in rats. To accomplish this, we measured several oxidative stress and apoptotic parameters to determine if indomethacin can induce gastric oxidative stress and if this stress could be treated by the use of *C. sempervirens* leave extract.

## Materials and methods

This study was carried-out on experimental animals and was performed in the Medical Physiology Department, Medical Research Division, National Research Centre, Egypt.

### Materials

Indomethacin and ranitidine (standard reference; where ranitidine is a standard treatment for indomethacin-related gastric ulcer) were purchased from Sigma Chemical Co. (St. Louis, MO, USA). All reagents were of analytical grade and were obtained from Biomeriéux company, France, through a local supplier.

### Plant material

*C. sempervirens* leaves were collected from the botanical garden of the National Research Center, Egypt, in October 2013. The plant was botanically identified and authenticated by Prof L. Boulos at the National Research Centre, Cairo, Egypt. Voucher specimen of each plant was deposited at the herbarium of the National Research Centre. The leaves were crushed, pulverized, and weighed in sequence to prepare the extraction.

### Preparation of the ethanolic extract

*C. sempervirens* leaves (5 kg) were air-dried in an oven at 40 °C for 4 days and then the dried leaves were cut and pulverized. They were then subjected to size reduction to get coarse powder of desired particle size. The powdered material was subjected to a successive extraction in a Soxhlet apparatus using solvent petroleum ether (40–60 °) and ethyl alcohol. The temperature was maintained on an electric heating mantel with a thermostat control. The extract was then concentrated to 3/4^th^ of its original volume using a rotavap apparatus. The concentrated extract was transferred to a *Petri dish* and evaporated on a thermostat-controlled water bath till powder was formed. This powder was vacuum dried in a desiccator till it was free of moisture. The extract was subjected to chemical tests for the detection of phyto-constituents. The final extract powder = 500 g. The extraction ratio was 1:10 and the extraction process followed the method of Chopra *et al*. (1986). The extraction process took one month from collection of the plant leaves till the final ethanolic extract was obtained.

### Thin layer chromatography (TLC) for isolation of leave extract cupressuflavone

TLC was used to resolve and isolate the cupressuflavone constituent from *C. sempervirens* leave extract. Precoated silica gel plates (20 × 20 cm, Brinkman Silplate F-22, with fluorescent indicator, 0.25, 0.5, or 2.0 mm gel thickness) were used in all separations. With these plates, the substituted flavonoid present was easily detected as yellow or golden bands by viewing the developed plates under long-wave ultraviolet (UV) light. Cupressuflavone was isolated by applying the extract as bands to TLC plates, developed in appropriate solvent systems, locating the compound under UV light, and then scraping and eluting with chloroform or acetone followed. The compound obtained was crystallized directly from appropriate solvents (usually ether or ether-hexane), or if necessary, the compound was subjected to further cleanup by TLC. The solvent systems used and the numbers used to designate them are as follows: 1, methylene chloride; 2, chloroformethyl acetate (2:1); 3, methylene chloride-ether (2:1); 4, benzene-ether (20:1); 5, hexane-ethyl acetate-methanol (551); and 6, methylene chloride-ether (1:l) (Navickiene *et al*., 2001). This process took two months from ethanolic extract till isolation of cupressuflavone powder. The isolation ratio = 1:5, where 60 g of cupressuflavone ingredient was obtained from 300 g of the extract.

### Test for purity, quality and stability of the extract and cupressuflavone

Purity tests (microbiological, pesticide residues, heavy metals, radioactive residues, chemical, foreign organic matter and sulfated ash) were performed in accordance with Egyptian accepted protocol requirements and accredited to ISO/IEC 17025 consulting WHO guidelines on stability and quality controls of methods for medicinal plants (Farnsworth, 2001; Leung & Foster, 1996). The *C. sempervirens* leave extract and cupressuflavone were stored at cooling (–4 °C) in a tightly sealed container, protected from heat and light to prevent loss of the extract solvent and entry of water. *C. sempervirens* extract or cupressuflavone was dissolved in 1ml distilled water and given orally to each individual rat.

### Animals

Fifty-six male albino rats (120–125 g) of *Sprague Dawley* strains were obtained from the animal house of the National Research Centre, Egypt, and were kept in special plastic cages. The animals were maintained on a commercial balanced diet and tap water. The experiments were performed after approval from the Ethics Committee of the National Research Centre, Egypt, and in accordance with recommendations for proper care and use of laboratory animals (NIH publication no. 85:23 revised 1985).

### Experimental design

The lethal dose fifity (LD_50_) of *C. sempervirens* extract was determined previousely: 800 mg/kg bwt (Koriem, 2009), while 5%, 10% & 20% of the LD_50_ of the plant were found to be safe (Koriem *et al*., 2010). A pilot dose-response study included 15 gastric ucler rats pre-treated with 5%, 10% and 20% of the LD_50_ of *C. sempervirens* leaves extract 1 h prior to indomethacin administration. Macroscopic evaluation of gastric lesions was recorded and the obtained data were used to organize the experimental design of the study.

Fifty-six rats were divided into seven groups (8 rats each) as follows: **1^st^ group**: administered orally saline solution (1ml/ rat). **2^nd^ group**: orally administered indomethacin (25 mg/kg bwt). **3^rd^ group**: orally administered ranitidine (150 mg/kg bwt) 1 h prior to indomethacin oral administration. **4^th^ group**: received orally 5% LD_50_ of *C. sempervirens* leaves 1 h before indomethacin oral administration. **5^th^ group**: received orally10% LD5_0_ of *C. sempervirens* leaves 1 h prior to indomethacin oral administration. **6^th^ group**: received orally 20% LD_50_ of *C. sempervirens* leaves 1 h before indomethacin oral administration. **7^th^ group**: received orally cupressuflavone (200mg/kg bwt) 1 h prior to indomethacin oral administration.

After 6 h, all animals were sacrificed using a dose of diethyl ether.

### Macroscopic analysis of stomach tissue

The rat stomach, subjected to macroscopic evaluation to record gastric lesions, was opened along the greater curvature and washed with physiological saline solution. The ulcer rate was then determined macroscopically. The area size was determined using millimeter filter paper and magnifier.

### Stomach tissue preparation

The animals were decapitated and then dissected, whereby the stomach tissues were obtained, washed in cold saline, and dried between filter papers. They were weighed, homogenized and kept at –80 °C for further investigation; 0.5 g of stomach tissue was dissolved in 2.5 ml of Tris buffer solution, then homogenized in a homogenizer for exactly 30 min. Then the stomach tissues were centrifuged for exactly 20 min at 7 000 × *g*, the supernatants were thus separated and used for determination of antioxidant activities.

### Determination of gastric biochemical variables

Glutathione S-transferase (GST) level was assayed according to the method of Mannervik and Guthenberg (1981) with slight modification. Glutathione peroxidase (GPx) activity was determined according to the method of Pagalia and Valentine (1967). Gastric catalase (CAT) activity assay was performed according to the method described by Aebi (1984). Determination of reduced glutathione (GSH) level was carried out according to the method of Ellman (1959), with slight modification. Evaluation of gastric glutathione reductase (GR) level was done according to the method of Goldberg and Spooner (1983). Gastric lipid peroxidation (MDA) level, which is a measure of lipid peroxidation, was measured by the method of Okhawa *et al*. (1979). Superoxide dismutase (SOD) activity was determined on the basis of the method by Suttle (1986). Gastric total protein was measured according to the method of Gornall *et al*. (1949). Protein carbonyl (PC) level was determined using the method described by Levine *et al*. (1990).

### Immunohistochemical detection of *p53* and bcl-2 expression in stomach tissue

#### Principle

Immunohistochemical (IHC) staining technique allows visualization of antigens via sequential application of a specific antibody to the antigen (primary antibody), a secondary antibody to the primary antibody and an enzyme complex with a chromogenic substrate with interposed washing steps. The enzymatic activation of the chromogen results in a visible reaction product at the antigen site. The specimen may then be counterstained and overslipped. Results are interpreted using a light microscope (Omata *et al*., 1980; Tweddle *et al*., 2001).

#### Staining protocol

Staining procedure for *p*53 and bcl-2 was performed according to Hsu & Raine (1984) and Elias *et al*. (1989) as follows: gastric tissue sections were cut and mounted on positive slides coated with a suitable tissue adhesive. Sections were de-paraffinized in xylen, then the tissue sections were re-hydrated through graded ethyl alcohols. Endogenous peroxidase was neutralized by using 1 ml 30% hydrogen peroxide to 9 ml absolute methanol, then the slides were submerged in peroxidase solution for 10 min. The slides were washed with phosphate buffer solution (PBS) for 2 min. Primary antibody (100 μl) was applied to each section completely covering the tissue, then the slides were incubated for 30 min and finally rinsed with PBS for 2 min. Another secondary antibody (100 μl) was applied to each section completely covering the tissue; then the slides were incubated for 20 min and rinsed with PBS for 3 min. Additional 100 μl of enzyme conjugate was applied to each section to completely cover the tissue, then the slides were incubated for 10 min and rinsed with PBS for 2 min. Chromogen was added and the slides were rinsed in water, then counterstained with hematoxylin. The slides were dehydrated, cleared and the sections were investigated under a microscope.

### Statistical analysis

The results were expressed as mean ± standard error (SE). Statistical significance was determined through one-way analysis of variance (ANOVA), using the SPSS 13 software package for Windows (Chicago, IL). Post hoc testing was performed for inter-group comparisons using the least significance difference (Tukey) test; p-values less than 0.05 were considered statistically significant. *p≤0.05 significant difference compared to control and **p≤0.01 highly significant difference compared to control.

## Results

In the present study, no differences were observed in body weight, food or water intake during the experimental period.

**Figure 1:**
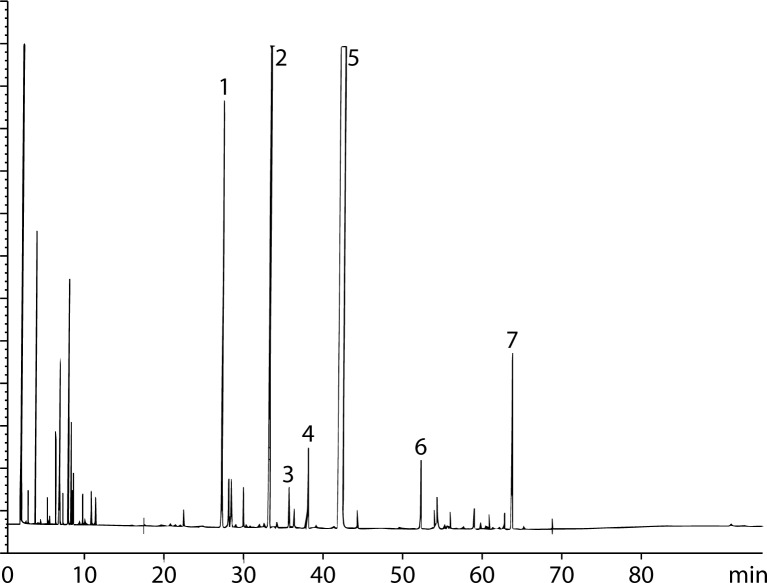
Chromatographic profile of *C. sempervirens* leaves extract showing different ingredients, where 1 is amenoflavone; 2 is rutin; 3 is myricitrin; 4 is anthocyanidin; 5 is cupressuflavone; 6 are tannins; 7 is quercetin.

Figure 1 shows the results of chromatographic analysis, demonstrating the constituents of *C. sempervirens* leaves extract as follows: cupressuflavone (67.2%), amenoflavone (4.5%), flavonoids (rutin, quercetin and myricitrin; 5.1%, 3.9% and 2.1% respectively), anthocyanidin (3.8%) and tannins (ellagic acid, gallic acid, phenyl isopropanoids, caffeic acid, coumaric acid, ferulic acid; 2.0%, 2.6%, 1.4%, 3.2, 2.8, 1.4%, respectively).

**Figure 2:**
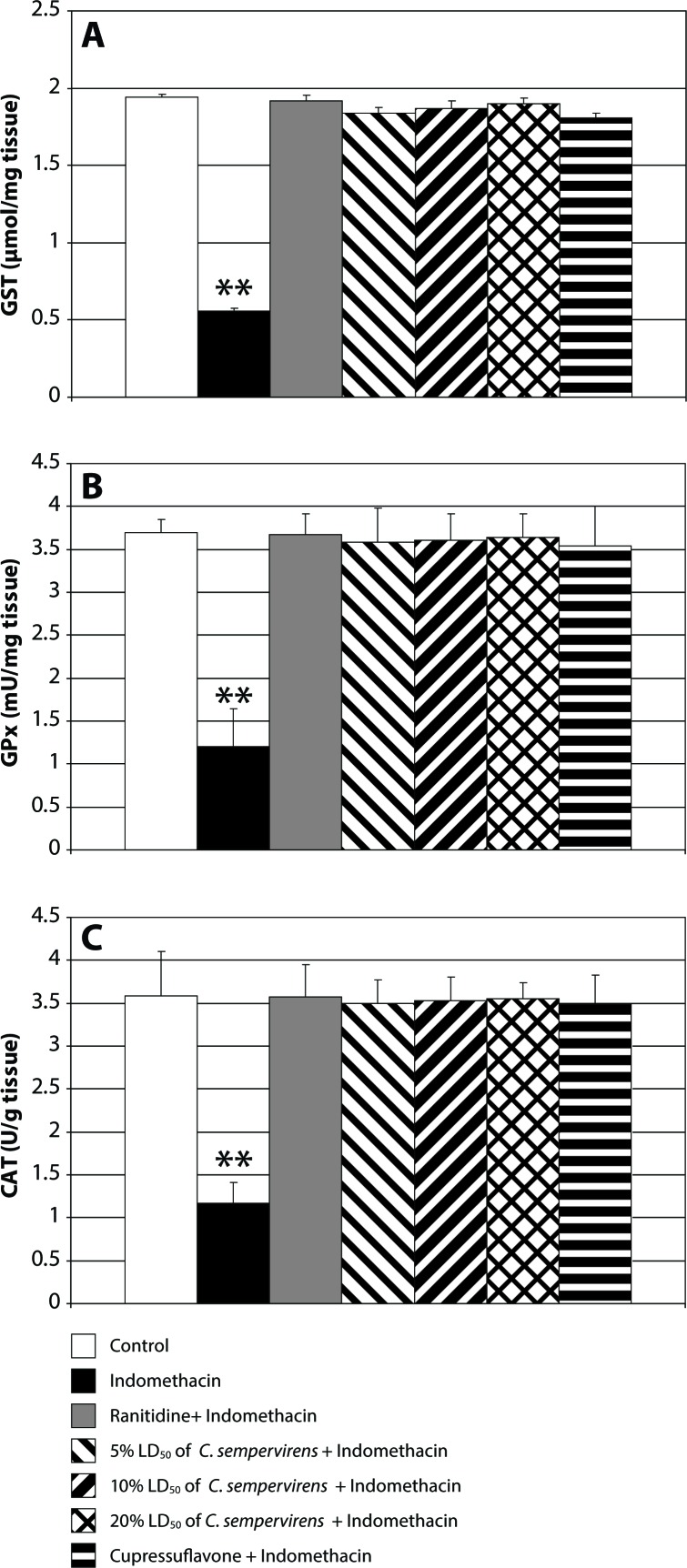
Effect of *C. sempervirens* and cupressuflavone on GST (**A**), GPx (**B**) and CAT (**C**) levels in gastric tissues. Results were expressed as mean ± SE and significant difference to control group at *p*≤0.05. ANOVA showed a highly significant difference between all groups at *p*≤0.0001. **p≤0.01 highly significant difference compared to control.

Figure 2 presents gastric GST, GPx and CAT in control, indomethacin-, ranitidine+ indomethacin-, *C. sempervirens* + indomethacin- and cupressuflavone + indomethacin-treated animals. Compared to the control group, the indomethacin-treated animals had significantly lower GST, GPx and CAT levels in stomach tissues. However, treatment of the animals with ranitidine, *C. sempervirens* or cupressuflavone increased significantly the gastric GST, GPx or CAT levels in the indomethacin-exposed animals. *C. sempervirens* extract at the highest dose was more potent than at the two lower doses. *C. sempervirens* leaves extract was more effective than cupressuflavone, while ranitidine was more potent than *C. sempervirens* extract and cupressuflavone.

**Figure 3:**
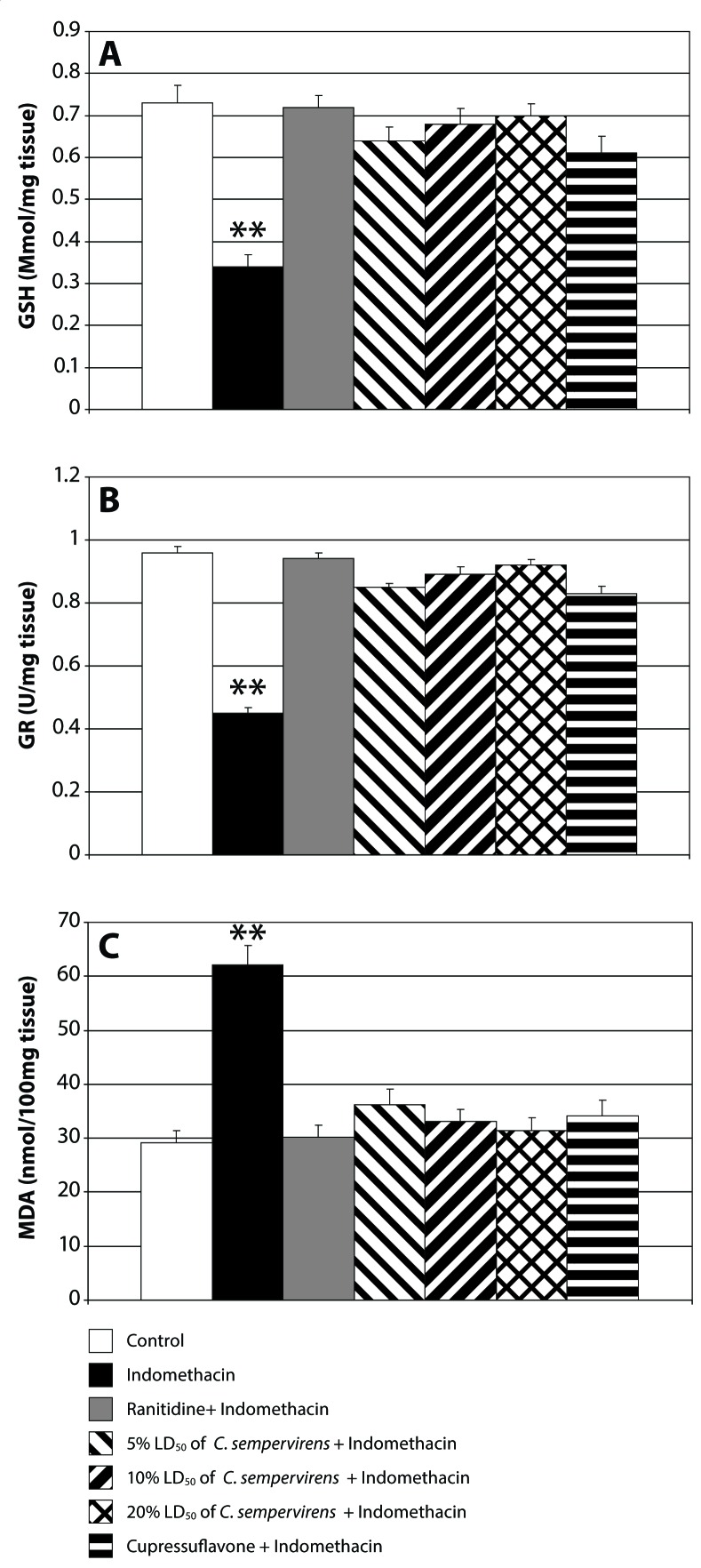
Effect of *C. sempervirens* and cupressuflavone on GSH (**A**), GR (**B**) and MDA (**C**) levels in gastric tissues. Results were expressed as mean ± SE and significant difference compared to control group at *p*≤0.05. ANOVA showed a highly significant difference between all groups at *p*≤0.0001. **p≤0.01 highly significant difference compared to control.

Figure 3 shows stomach GSH, GR and MDA in control, indomethacin-, ranitidine+ indomethacin-, *C. sempervirens* + indomethacin- and cupressuflavone + indomethacin-treated animals. Indomethacin induced a significant decrease in antioxidant enzyme levels of GSH and GR, while there is a significant increase in lipid peroxidation by increasing the level of malondialdehyde (MDA). The animals treated by ranitidine, *C. sempervirens* or cupressuflavone show a significant increase of GSH and GR levels and a decreasing lipid peroxidation level compared to the animals in the indomethacin only treated group. On the other hand, the highest dose of *C. sempervirens* extract was more potent than the lower two doses. *C. sempervirens* leaves extract was more effective than cupressuflavone, while ranitidine was more potent than *C. sempervirens* extract and cupressuflavone.

**Figure 4:**
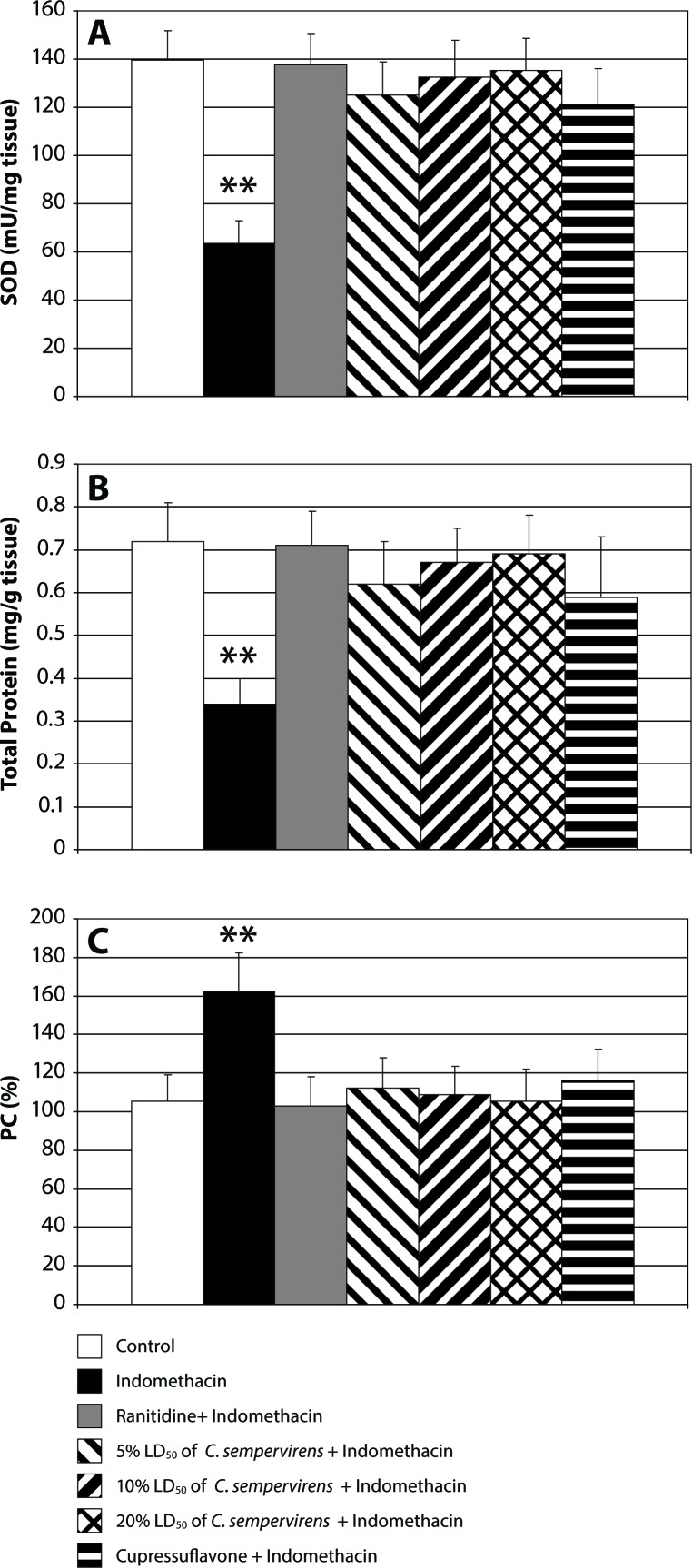
Effect of *C. sempervirens* and cupressuflavone on SOD (**A**), total protein (**B**) and PC (**C**) levels in gastric tissues. Results were expressed as mean ± SE and significant difference compared to control group at *p*≤0.05. ANOVA showed a highly significant difference between all groups at *p*≤0.0001. **p≤0.01 highly significant difference compared to control.

Figure 4 reveals that indomethacin-treated animals have a significantly lowered SOD and total protein level in the gastric tissue, while the stomach PC level is significantly increased after indomethacin administration. However, treatment of the animals with ranitidine, *C. sempervirens* or cupressuflavone increased the SOD and total protein levels significantly. Yet *C. sempervirens* extract at the highest dose was more potent than the lower two doses. *C. sempervirens* leaves extract was more effective than cupressuflavone, while ranitidine was more potent than *C. sempervirens* extract and cupressuflavone.

**Figure 5:**
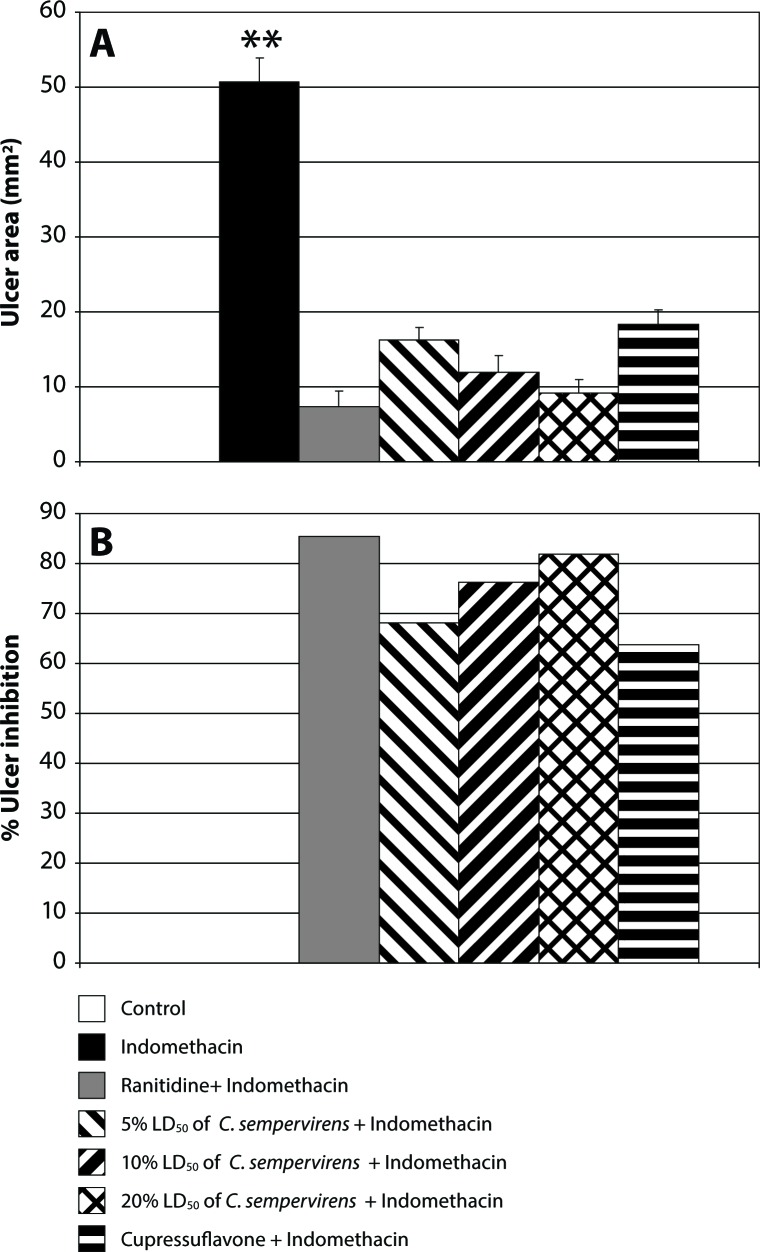
Effect of *C. sempervirens* and cupressuflavone on ulcer area (**A**) and percentage of ulcer inhibition (**B**) in gastric tissues. Results were expressed as mean ± SE and significant difference compared to control group at *p*≤0.05. ANOVA showed a highly significant difference between all groups at *p*≤0.0001. **p≤0.01 highly significant difference compared to control.

Figure 5 displays the effect of ranitidine, *C. sempervirens* and cupressuflavone on the ulcer area in gastric tissue. Ranitidine clearly inhibited the ulcer area by 85.5%, while inhibition of the ulcer area by *C. sempervirens* doses ranges from 68.1% to 81.9%. Cupressuflavone reduced gastric ulcer by 63.8%.

**Figure 6:**
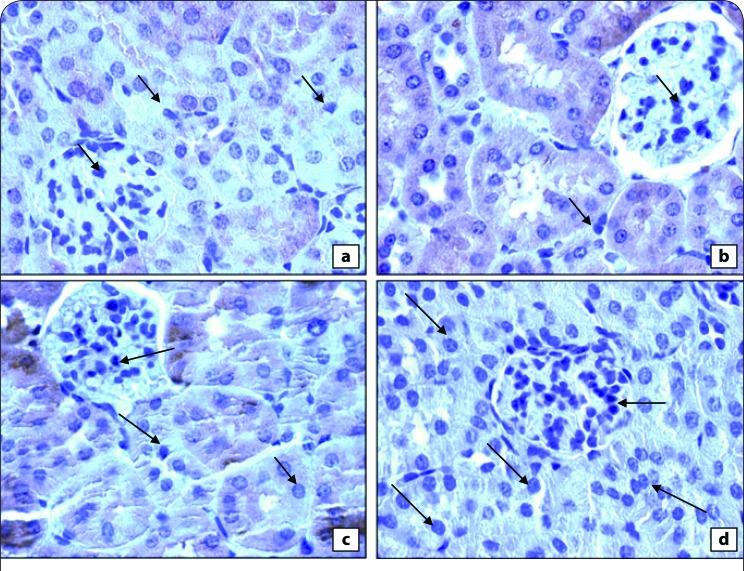
**(a)** Immunohistochemically-stained stomach tissue sections showing apoptotic marker *p*53 expression in control rats with normal amount of expressed *p*53. **(b)** Tissue sections showing the effect of indomethacin on apoptotic marker *p*53 expression, indomethacin decreased strongly *p*53 expression in stomach sections as evidenced by weak immunostaining in the rat stomachs. **(c)** There was an increase in expression of *p*53 in the rat stomach treated with cupressuflavone, evident by the positive staining **(d)** Expression of *p*53 was increased markedly on administration of the highest dose of *C. sempervirens*, as well evident by the positive staining.

Figure 6 shows the photomicrographs of immunohistochemically stained stomach tissue sections. Saline administration resulted in a normal amount of expressed *p*53 (Figure 2A). Yet the amount of *p*53 was potentially decreased in indomethacin-administrated rats (Figure 2B), as compared to control rats. The treatment of indomethacin-administered rats with *C. sempervirens* or cupressuflavon increased the amount of expressed *p*53 (Figures 2C & D), as compared to indomethacin-administered rats.

**Figure 7:**
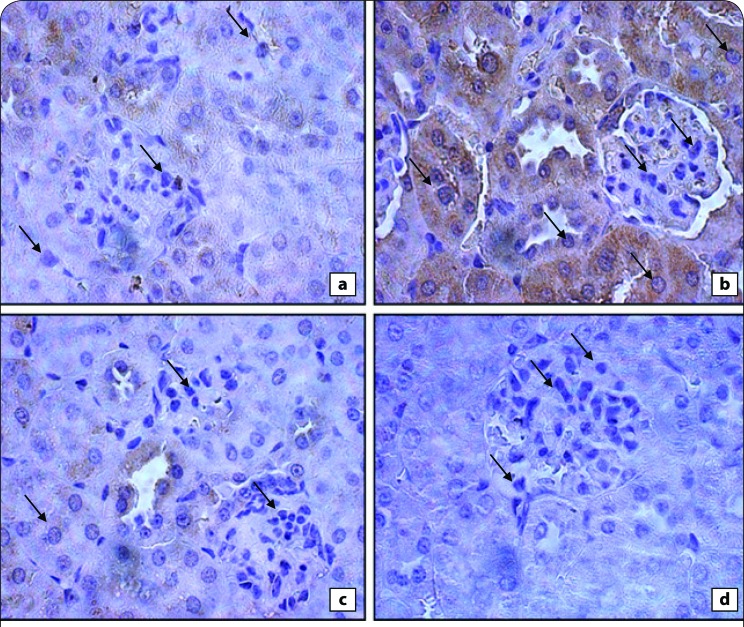
**(a)** Immunohistochemically-stained stomach tissue sections showing bcl-2 expression in control group with practically normal amount of expressed bcl-2 **(b)** indomethacin administration strongly induced bcl-2 expression in stomach sections as evidenced by strong immunostaining **(c)** inhibition of bcl-2 expression in the rat stomach treated with cupressuflavone, as evidenced by weak immunostaining **(d)** higher dose of *C. sempervirens* treatment showed lower expression, implying that bcl-2 was inhibited, as evidenced by weaker immunostaining.

Figure 7 exhibits the photomicrographs of immunohistochemically stained stomach tissue sections. Normal rats showed a normal amount of expressed bcl-2 (Figure 3A), while the amount of bcl-2 was potentially increased in indomethacin-administered rats (Figure 3B). Treatment of indomethacin-administered rats with *C. sempervirens* or cupressuflavon potentially decreased the elevated amount of expressed bcl-2 (Figures 3C & D) compared to the indomethacin-treated group.

## Discussion

The mechanism of antiulcerogenic protection relies on cellular action of flavonoid ingredients of the plant and their ability to modulate gene expression and the activity level of enzymes involved in antioxidant defense and glutathione activity (Wiegand *et al*., 2009).

The aim of this study was to prove that pretreatment with *C. sempervirens* extract could modulate indomethacin-induced gastric oxidative stress and ameliorate tissue damage in rats.

In this study, *in vivo* oxidative effect of indomethacin on gastric tissues of albino rats was established. We also studied the potential antioxidant and anti-apoptotic activities of *C. sempervirens* to prevent oxidative stress-related damage to gastric tissues. The results obtained indicated that *C. sempervirens* treated oxidative damage, possibly by scavenging existing ROS, while halting the production of further ROS, nitric oxide, lipid peroxidation, and protein carbonylation. In addition, *C. sempervirens* increased the levels of GSH and SOD as well as the activity of the detoxification enzyme GPx. At the highest dose, *C. sempervirens* was more potent than at the lower two doses.

Indomethacin-exposed animals showed significant decrease in GSH in the stomach tissue tested, indicating that exposure to indomethacin induced oxidative stress in these tissues. *C. sempervirens* was capable to restore GSH levels in these tissues. A possible explanation for the decrease in GSH levels is the reduction in enzymatic activities involved in GSH synthesis and/or oxidation of GSH to GSSG under oxidative stress. An alternative explanation could be an increase in extra-cellular levels of glutamate, which in turn might inhibit the uptake of cystine via the glutamate-cystine exchange system, thereby reducing GSH synthesis. Our results are in line with the findings reported by Emami *et al*. (2007) and Ibrahim *et al*. (2007) who found that fruits of all species of *C. sempervirens* possessed antioxidant activity when tested either with the ferric thiocyanate (FTC) or the thiobarbituric acid (TBA) method.

In the current study, indomethacin induced a significant increase in MDA levels in stomach tissues of the animals, as compared to the *C. sempervirens*-treated group, thus pointing to the antioxidant role of *C. sempervirens* in protecting animals from indomethacin-induced damage. Our results are in accordance with the previously reported increase in lipid peroxidation in response to indomethacin (Miura *et al*., 2002; Pennington & Smith, 1978). Concomitant reduction of GSH levels might have hampered the decomposition of lipid peroxides in indomethacin-treated animals. *C. sempervirens* was able to prevent lipid peroxidation by supplying an adequate amount of GSH as a substrate for glutathione peroxidase to effectively decompose lipid peroxides in mice and thus to reduce MDA levels.

Gastric total protein and protein carbonylation determined in this study are reliable parameters of oxidative stress, lacking interference from other non-protein substances (Esterbauer *et al*., 1991) and measuring carbonylation of various protein residues, including lysine and arginine. Our results showed that stomach total protein was decreased while protein carbonyl was increased in indomethacin-treated animals, yet *C. sempervirens* prevented these changes. The results are in line with those reported previously (Moon *et al*., 2006; Sivaramakrishnan *et al*., 2008).

The increase in lipid peroxidation was accompanied by a concomitant decrease in the activity of antioxidant enzymes, such as SOD and GR. SOD is an enzyme responsible for the detoxification of highly reactive and potentially toxic radicals to less toxic hydrogen peroxide (Fridovich, 1983). The results of the present study indicated an inhibition of the enzyme in the stomach of indomethacin-treated animals. These results are in agreement with those of Moon *et al*. (2006) and Sivaramakrishnan *et al*. (2008) who suggested that plant-derived flavonoids can increase SOD and GPx level and exert anti-oxidative action and achieve beneficial health effects.

The significant reduction in the activity of GPx observed after indomethacin administration in this study may have been partially due to diminished levels of GSH, which needs GPx as substrate. Our results are also in accordance with the previously reported decrease in GPx activity after indomethacin administration due to the interaction of indomethacin with the essential selenocysteine moiety of the enzyme (Yin *et al*., 2014; Odabasoglu *et al*., 2006).

Increased CAT activity was observed in animals administered indomethacin. *C. sempervirens* pretreatment, yet again, reversed CAT activity in the indomethacin-treated group. Changes in CAT activity were observed in the stomach tissues that were undergoing oxidative stress. Increased CAT activity in indomethacin-treated animals could be an adaptive response to the higher levels of H_2_O_2_ generated by inhibition of GPx. The response of this antioxidant enzyme to oxidative agents is tissue/ organ specific and has an adaptive character. Increased CAT activity in indomethacin-treated animals could be an adaptive response to the higher levels of H_2_O_2_ generated by inhibition of GPx. A possible mechanism for the restored CAT activity in indomethacin-exposed rats treated with *C. sempervirens* may be the scavenging of free radicals by the amide antioxidant ingredient or by providing more GSH, which is a substrate for GPx.

In the current study, anti-apoptotic activity of *C. sempervirens* was also recorded. *P53* and bcl-2 are closely related to the majority of human toxicities and cancer (Gu *et al*., 2009). The apoptotic marker *p*53 is a critical regulator of apoptosis in many cells. It stimulates a wide network of signals that act either through extrinsic or intrinsic pathways of apoptosis (Yerlikaya *et al*., 2012) by activating the transcription of downstream genes such as p21 and Bax to induce the apoptotic process, thus inhibiting the growth of cells with damaged DNA. On the other hand, bcl-2 was reported to function primarily by blocking the apoptosis pathway (Hussein & Ahmed, 2010). The Bcl-2 gene product is a negative regulator of apoptosis, which forms a hetero-dimer complex with Bax and neutralizes the effect of pro-apoptosis (Chaudhary *et al*., 2012). Our results indicated that indomethacin decreased *p*53 expression while increasing bcl-2 expression. On the other side, administration of *C. sempervirens* to indomethacin-exposed rats increased *p*53 expression while it decreased bcl-2 expression. These results were in agreement with those of Ye *et al*. (2009) who found that *C. sempervirens* averted liver toxicity induced by indomethacin. The mechanism of protection depends on flavonoids, which act as phyto-estrogens inducing aromatase activity in cells and increased mRNA expression with concurrent elevation of the use of promoters I.3/II. Several protein kinases are activated (PKC, P38, ERK-1/2) and consequently the transcriptional factor CREB is ultimately activated in gene regulation and protein synthesis is consequently increased. These data coincide with the findings of Rizk *et al*. (2007) and Ibrahim *et al*. (2007) who reported that *C. sempervirens* leaves extract exhibited protection against CCL_4_-induced lethality in rats, suggesting a protective action by prevention of CCL_4_-induced toxicity.

In conclusion, the results reported here indicated that *C. sempervirens-pretreated* rats exhibited enhanced lipid peroxidation accompanied by decrease in the antioxidant capacity of the cell. The decreased antioxidant ability of the cell results in reduced disposal of oxygen-free radicals and peroxides that may exceed the cellular defense system and eventually cause apoptosis and cellular injury by damaging DNA structure, proteins, and lipids and may thus ultimately be responsible for gastric ulcer. The suppressive effect of *C. sempervirens* on indomethacin-related effects appears to be through enhancement of endogenous antioxidant enzymes and disposal of free radicals as well as anti-apoptotic activity of *C. sempervirens,* which proved more potent at the highest dose applied.

## References

[CIT0001] Aebi H (1984). Catalase *in vitro*. Method Enzymol.

[CIT0002] Agrawal PK (1989). Carbon-13 NMR of Flavonoids.

[CIT0003] Ames BN, Shigenaga MK, Hagen TM (1993). Oxidants, antioxidants and the degenerative diseases of aging. Proc. Natl. Acad. Sci.

[CIT0004] Chaudhary SC, Siddiqui MS, Athar M, Alam MS (2012). D-Limonene modulates inflammation, oxidative stress and Ras-ERK pathway to inhibit murine skin tumorigenesis. Hum Exp Toxicol.

[CIT0005] Chevallier A (1996). The Encyclopedia of Medicinal Plants Dorling Kindersley.

[CIT0006] Chiej R (1984). Encyclopaedia of medicinal plants.

[CIT0007] Chopra RN, Nayar SL, Chopra IC (1986). Glossary of Indian Medicinal Plants (Including the Supplement).

[CIT0008] Das D, Bandyopadhyay D, Bhattacharjee M, Banerjee RK (1997). Hydroxyl radical is the majorcausative factor in stress-induced gastric ulceration. Free radical Biol Med.

[CIT0009] Duke JA (2002). Handbook of Medicinal Herbs.

[CIT0010] Elias JM, Margiotta M, Gabore D (1989). Sensitivity and detection efficiency of the peroxidase antiperoxidase (PAP) avidin-biotin peroxidase complex (ABC), and peroxidase-labelled avidin-biotin (LAB) methods. Am J Clin Pathol.

[CIT0011] Ellman GL (1959). Tissue sulphydryl groups. Arch Biochem Biophys.

[CIT0012] Emami SA, Asili J, Mohagheghi Z, Hassanzadeh MK (2007). Antioxidant activity of leaves and fruits of iranian conifers. Avid Based Compl Altern Med.

[CIT0013] Esterbauer H, Schaur RJ, Zollner H (1991). Chemistry and biochemistry of 4-hydroxynonenal, malonaldehyde and related aldehydes. Free Radic Biol Med.

[CIT0014] Farnsworth NR (2001). NAPRALERT Database.

[CIT0015] Fridovich I (1983). Superoxide radical as an endogenous toxicant. Ann Rev Pharmacol Toxicol.

[CIT0016] Goldberg DM, Spooner RJ, Bergmeyen H. V (1983). In Methods of enzymatic analysis.

[CIT0017] Gornall AC, Bardawill CJ, David MM (1949). Determination of serum proteins by means of the Biuret-reaction. J Biol Chem.

[CIT0018] Gu Q, Hu C, Chen Q, Xia Y, Feng J, Yang H (2009). Development of a rat model by 3, 4-benzopyrene intra-pulmonary injection and evaluation of the effect of green tea drinking on *p*53 and bcl-2 expression in lung carcinoma. Cancer Detect Prev.

[CIT0019] Harborne JB (1993). The Flavonoids Advaces in Research.

[CIT0020] Hortus EZ (1976). Liberty Hyde Bailey Hortorium.

[CIT0021] Hsu SM, Raine L (1984). In Advances in Immunochem DeLellis.

[CIT0022] Hussein AM, Ahmed OM (2010). Regioselective one-pot synthesis and antiproliferative and apoptosis effects of some novel tetrazolo[1, 5-a]pyrimidine derivatives. Bioorg Med.

[CIT0023] Ibrahim NA, El-Seedi HR, Mohammed MM (2007). Phyto-chemical investigation and hepato-protective activity of *Cupressus sempervirens* L. leaves growing in Egypt. Natl Prod Res.

[CIT0024] Isenberg JI, McQuaid KR, Laine L, Walsh JH, Lippincott J. B (1995). Acid peptic disorders.

[CIT0025] Kassem FF, Harraz FM, El-Sebakhy N A, De Pooter H L, Schamp N M, Abu-Shleib H (1991). Composition of the essential oil of Egyptian Cupressus sempervirens L. cones. Flavour Fragr J.

[CIT0026] Koriem KMM (2009). Lead toxicity and the protective role of *Cupressus sempervirens* seeds growing in Egypt. Rev Latinoamer. Quím.

[CIT0027] Koriem KM, Arbid MS, El-Gendy NF (2010). The protective role of *Tropaeolum majus* on blood and liver toxicity induced by diethyl maleate in rats. Toxicol Mech Methods.

[CIT0028] Leung AY, Foster S (1996). Encyclopedia of common natural ingredients used in food, drugs and cosmetics.

[CIT0029] Levine RL, Garland D, Oliver CN, Amici A, Climent I, Lenz AG, Ahn BW, Shaltiel S, Stadtman ER (1990). Determination of carbonyl content in oxidatively modified proteins. Method Enzymol.

[CIT0030] Mannervik B, Guthenberg C (1981). Glutathione transferase (human placenta). Methods Enzymol.

[CIT0031] Miura T, Muraoka S, Fujimoto Y (2002). Lipid peroxidation induced by indomethacin with horseradish peroxidase and hydrogen peroxide: involvement of indomethacin radicals. Biochem Pharmacol.

[CIT0032] Moon HK, Yang ES, Park JW (2006). Protection of peroxynitrite-induced DNA damage by dietary antioxidants. Arch Pharmacol Res.

[CIT0033] Navickiene HMD, Lopes LMX (2001). Alkamides and phenethyl derivatives from Aristolochia gehrtii. J Braz Chem Soc.

[CIT0034] Odabasoglu F, Cakir A, Suleyman H, Aslan A, Bayir Y, Halici M, Kazaz C (2006). Gastroprotective and antioxidant effects of usnic acid on indomethacin-induced gastric ulcer in rats. J Ethnopharmacol.

[CIT0035] Okhawa H, Ohishi N, Yagi K (1979). Assay of lipid peroxides in animal tissue by thiobarbituric acid reaction. Anal Biochem.

[CIT0036] Omata M, Liew C. T, Asheavai M, Peters RL (1980). Nonimmunologic binding of horseradish peroxidase to hepatitis B surface antigen: A possible source of error in immunohistochemistery. Am J Clin Pathol.

[CIT0037] Pagalia DE, Valentine WN (1967). Studies on the quantitative and qualitative characterization of erythrocyte glutathione peroxidase. J Lab Clin Med.

[CIT0038] Pennington SN, Smith CP (1978). Indomethacin stimulation of lipid peroxidation and chemilumine-scense in rat liver microsomes. Lipids.

[CIT0039] Rizk MA, Abdallah MS, Sharara HM, Ali SA, Ibrahim NA, Mostafa MM (2007). Efficiency of Cupressus sempiverenes L.and Juniperus Phoenicea against carbon tetrachloride hepatotoxicity in rats. Trends Med Res.

[CIT0040] Said OS, Khalil M, Fulder H (2002). Ethnopharmacological survey of medicinal plants in Israel, the Golan heights and the west Bank region. J Ethanopharmacol.

[CIT0041] Sivaramakrishnan V, Narasimha P, Shilpa M, Kumar VR, Devaraj SN (2008). Attenuation of N-nitrosodiethylamine-induced hepatocellular carcinogenesis by a novel flavonol Morin. Chem Biol Inter.

[CIT0042] Suttle NF (1986). Copper deficiency in ruminants; recent developments. Veter Record.

[CIT0043] Toma W, Gracioso J, Andrade FD, Hiruma-Lima CA, Vilegas W, Souza BA (2002). Antiulcerogenic activity of four extracts obtained from the bark wood of *Quassia amara* L. (Simaroubaceae). Biol Pharm Bull.

[CIT0044] Tweddle DA, Malcolm AJ, Cole M, Pearson AD, Lunee J (2001). *p*53 cellular localization and function in neuroblastoma. Am J Pathol.

[CIT0045] Uncini MRE, Camangi K, Tomei PE (2001). Curing animals with plants: traditional usage in Tuscany (Italy). J Ethnopharmacol.

[CIT0046] Uphof JCT (1959). Dictionary of Economic Plants.

[CIT0047] Usher GA (1974). Dictionary of Plants Used by Man.

[CIT0048] Vidakovic M (1991). Morphology and Variation. Annales forestales. Analiza sumar.

[CIT0049] Waisel Y, Epstein V (2000). How to reduce air pollution by Cupressus pollen? Allerg. Immunol (Paris).

[CIT0050] Wiegand H, Wagner AE, Boesch-Saadatmandi C (2009). Effect of dietary genistein on Phase II and antioxidant enzymes in rat liver. Can Gen Prot.

[CIT0051] Ye F, Chen y, Hoang T, Montgomery RL, Zhao XH, Bu H, Hu T, Taketo MM, van-Es JH, Clevers H, Hsieh J, Bassel-Duby R, Olson EN, Lu QR (2009). HDAC1 and HDAC2 regulate oligodendrocyte differentiation by disrupting the β-catenin–TCF interaction. Nature Neurosci.

[CIT0052] Yerlikaya A, Okur E, Ulukaya E (2012). The *p*53-independent induction of apoptosis in breast cancer cells in response to proteosome inhibitor bortezomib. Tumor Biol.

[CIT0053] Yin H, Pan X, Song Z, Wang S, Yang L, Sun G (2014). Protective Effect of Wheat Peptides against Indomethacin-Induced Oxidative Stress in IEC-6 Cells. Nutrients.

